# Stability of SnSe-Based Thermoelectric Compounds

**DOI:** 10.3390/ma18184228

**Published:** 2025-09-09

**Authors:** Moritz Thiem, Ann-Katrin Emmerich, Iliya Radulov, Anke Weidenkaff, Wenjie Xie

**Affiliations:** 1 Materials and Resources, Institute of Materials Science, Technical University of Darmstadt, 64287 Darmstadt, Germany; moritz.thiem@mr.tu-darmstadt.de (M.T.); ann-katrin.emmerich@mr.tu-darmstadt.de (A.-K.E.); anke.weidenkaff@mr.tu-darmstadt.de (A.W.); 2 Fraunhofer Research Institution for Materials Recycling and Resource Strategies IWKS, 63457 Hanau, Germany; iliya.angelov.radulov@iwks.fraunhofer.de

**Keywords:** thermal stability, thermoelectric, SnSe

## Abstract

SnSe compounds are studied as promising candidates for thermoelectric (TE) applications, primarily due to their remarkable achievement of a high *ZT* value and the relative abundance of their constituent elements. In former studies, a significant disparity in the performance of polycrystalline SnSe compounds has been observed, and the reasons for the non-reproducibility have been investigated. This study focuses on the impact of sintering temperature on the thermoelectric properties of both Br-doped and undoped SnSe materials. Through a targeted synthesis approach, we achieved a *ZT* value of 1.04 at *T* = 873 K. The results reveal a critical challenge in controlling the mobility of ions and defects for long-term application of SnSe-based thermoelectric materials. The peak *ZT* values observed in the initial measurements are not sustainable, as the thermoelectric performance experiences a decline during multiple heating–cooling cycles. This issue is further underscored by extended annealing experiments, which resulted in a substantial *ZT* decrease of approximately 50%. These outcomes emphasise the need for a comprehensive understanding of the long-term stability of SnSe materials in thermoelectric applications. Additionally, they emphasise the importance of conducting heating–cooling measurements in thermoelectric systems, particularly when aiming to achieve and maintain high *ZT* values for longer periods.

## 1. Introduction

Today, research on sustainable energy converters is primarily driven by global climate change. The increase in global energy consumption necessitates the exploration of novel low-carbon energy sources [1,2]. Thermoelectric materials, which can directly convert “waste” heat into electricity, present a promising avenue for enhancing the sustainability of existing systems, including combustion engines, solar energy utilisation, and waste heat from various industrial processes [3,4,5,6]. Additionally, thermoelectric materials offer versatile applications, such as temperature regulation, refrigeration, and power generation [7,8,9]. To evaluate the effectiveness of these systems, the dimensionless thermoelectric figure of merit *ZT* = *σS^2^T*/(*κ_L_* + *κ_e_*) (where *σ* represents electrical conductivity; *S* signifies the Seebeck coefficient; *κ_e_* and *κ_L_* denote the contributions of electrons and lattice to the total thermal conductivity, *κ_tot_*; and *T* represents the absolute temperature) is employed.

Notably, for n-type single crystalline SnSe, *ZT* values as high as 2.8 have been reported [10]. Given their unsuitable mechanical properties, high production costs, and protracted synthesis processes [11], there has been a shift in focus towards applying polycrystalline SnSe. Recent research has achieved *ZT* values exceeding 3.1 for polycrystalline samples, surpassing single-crystalline counterparts [12]. However, *ZT* values for polycrystalline systems exhibit a wide range, with some falling close to or usually lower than those of single-crystalline SnSe compounds. This variability is primarily attributed to differences in thermal conductivity, which appears to be linked to material density [13]. Zhou et al. demonstrated that reducing the thermal conductivity of polycrystalline SnSe to match that of single crystals is feasible through the purification of starting materials and post-synthesis treatments, resulting in a significant reduction in thermal conductivity compared to untreated samples [12]. The role of Sn oxide in this context remains uncertain, as additional Sn-oxide content does not appear to worsen thermal conductivity [14].

**Figure 1:**
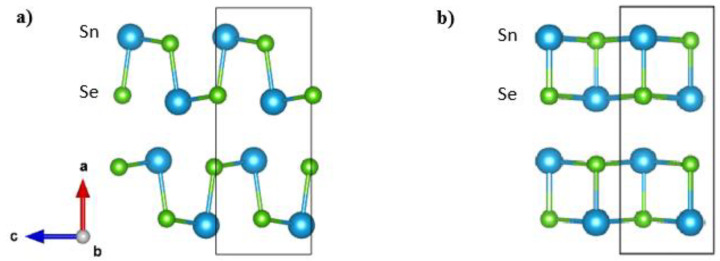
Crystal structures of SnSe in *Pnma* (**a**) and *Cmcm* (**b**) phases. Sn atoms are blue, and Se atoms are green. *Cmcm* crystal axes are chosen to match the *Pnma* phase.

High performance is only one requirement for the commercial application of thermoelectric materials; even more important is the reproducibility of that performance. Reproducibility remains a critical but largely missing aspect in the case of polycrystalline SnSe. Although there are some high *ZT* values reported for doped polycrystalline SnSe, only a few studies have been able to achieve *ZT* values larger than 2 [12,15,16,17]. In most studies in the literature, thermoelectric properties measurements and sintering were performed at temperatures well above 800 K [11,16,18,19,20,21,22,23,24,25,26,27]. It is worth mentioning that SnSe undergoes a phase transition at ~795 K < *T* < 810 K [28,29,30] from the *Pnma* to the *Cmcm* phase (Figure 1). Such a phase transition can result in potential damage to the microstructure of the sample during measurements, which is rarely discussed, and it may pose a concern for the long-term stability of SnSe compounds, especially since traversing the phase transition is known to cause microcracks [31,32].

To address the reproducibility and investigate whether sintering at different temperatures and measuring above the phase transition temperature affect material stability, we sintered samples at various temperatures, both below and above the phase transition, and measured their electrical and thermal transport properties in heating–cooling cycles. In this work, both n-type Br-doped SnSe_0.9_Br_0.1_ and undoped p-type SnSe were synthesised, sintered, and characterised. Our results indicate that the phase transition of SnSe can be used to explain low thermal conductivities in samples sintered above the phase transition, and it could also be responsible for the reported differences in density. Furthermore, our findings underscore the imperative nature of a reproducible, stable synthesis procedure and emphasise the need for stability tests in heating–cooling cycles of SnSe compounds.

## 2. Experimental Methods

### 2.1. Sample Preparation

SnSe and SnSe_0.9_Br_0.1_ synthesis starts by flame-sealing stoichiometric amounts of Sn shots (Chempur (Karlsruhe, Germany), 99.999% purity), Se shots (Chempur, 99.999% purity), and SnBr_2_ powder (Alfa Aesar (Haverhill, MA, USA), 99.2% purity) into a quartz tube under an argon atmosphere of 0.2 bar absolute pressure. Since the tube breaks during the cooling to room temperature, it was placed into an evacuated tube with a larger diameter. For the reaction, the temperature was heated up to 1223 K for 10 h and held at that temperature for 10 h. It was then air-cooled to room temperature. The obtained ingot was ground to a fine powder by ball milling (Pulverisette 7, Fritsch, Idar-Oberstein, Germany) in an argon atmosphere for a total of 3 h at 300 rpm with a sample-to-ball mass ratio of 1:10 in tungsten carbide (WC) balls and containers. Spark plasma sintering (SPS) was then performed with a “Dr. Sinter Lab SPS-211” machine by Fuji Electronic Industrial Co., (Tsurugashima, Saitama, Japan) on the ball-milled powder under a vacuum atmosphere. The powders were sintered in graphite dies with a diameter of 10 mm. A pressure of 50 MPa was applied with a sintering time of 5 min at different temperatures: 573 K, 673 K, 773 K, and 873 K for pure SnSe and 573 K, 673 K, 753 K, and 873 K for the Br-doped samples. The pressure was released immediately after the 5 min holding time. The final samples were of the following geometries: ~17 mm in thickness and 10 mm in diameter. The average achieved density was ~98% of the theoretical density of pure SnSe for all samples of all compositions. As for the later annealing procedure, previously cut sample pieces were wrapped in carbon paper and sealed in a quartz tube under an argon atmosphere. They were then kept in a furnace at 753 K for 336 h.

### 2.2. Structural Characterisation

The powder X-ray diffraction (XRD) patterns were all measured, using Mo-Kα1 radiation in a STOE STAD diffractometer (Darmstadt, Germany). Measurements were performed from 5° to 45° of 2*θ*, with a step size of 0.2° and a scan velocity of 0.4°/min. For analysis of phase composition and phase distribution, Scanning Electron Microscopy was used (SEM; TESCAN, VEGA3, Dortmund, Germany) with an energy-dispersive X-ray spectrometer (EDX; EDAX Genesis, AMTEK Gmbh, Unterschleissheim, Germany).

### 2.3. Transport Property Characterisation

For thermal transport property measurements, the thermal diffusivity, D, was measured with a Netzsch 467 HT HyperFlash Laser Flash Apparatus (LFA, Selb, Germany) on graphite spray-coated samples with thicknesses of about 1.2 mm. The thermal conductivity, *κ*, was then obtained by the following calculation: *κ* = *DdC_p_*, where *d* is the density and *C_p_* is the specific heat. *C_p_* was determined using the Dulong–Petit law, where *d* was measured using an Archimedes kit (KERN ALJ, KERN & Sohn Gmbh, Bahlingen-Frommern, Germany). For high-temperature measurements of the Seebeck coefficient, *S*, and electrical conductivity, *σ*, a ULVAC-RICO ZEM-3 device (Yokohama, Japan) with an R-type thermocouple was used under a helium atmosphere. The uncertainties for the characterisation techniques for thermoelectric properties are ±3% for *σ*, ±5% for *S*, and ±10% for *κ*. Measurements were performed in the temperature range of 373–873 K in steps of 50 K to 100 K. All transport properties were measured in the direction parallel to the pressing direction of the SPS (Figure S1).

### 2.4. Thermal Analysis

Differential Scanning Calorimetry (DSC; DSC 404 C, Netzsch, Selb, Germany) and Thermogravimetric Analysis (TGA; STA 409, Netzsch, Selb, Germany) were both performed under an argon atmosphere with a flow rate of 30 mL/min. For TGA, the sample chamber was flushed 2 h before each measurement to reduce the potential influence of residual air in the chamber. As for DSC, the sample chamber was pumped and flushed before the measurements to ensure an inert atmosphere. DSC measurement was performed in a range of 303–833 K in 10 K/min heating and cooling under an argon flow of 30 mL/min.

## 3. Results and Discussion

### 3.1. Phase and Microstructure

**Figure 2:**
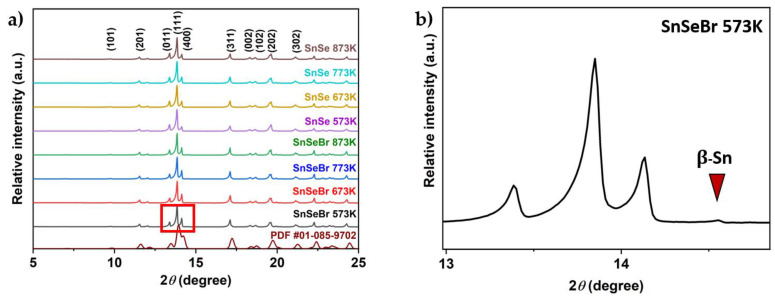
(**a**) Room-temperature XRD patterns of powder-sample SnSe and Br-doped SnSe (SnSe_0.9_Br) with a literature comparison [34]. (**b**) Close-up (Red box) of the β-Sn peak for a Br-doped sample sintered at 573 K (SnSeBr 573K). The number after each sample name is the sintering temperature.

**Figure 3:**
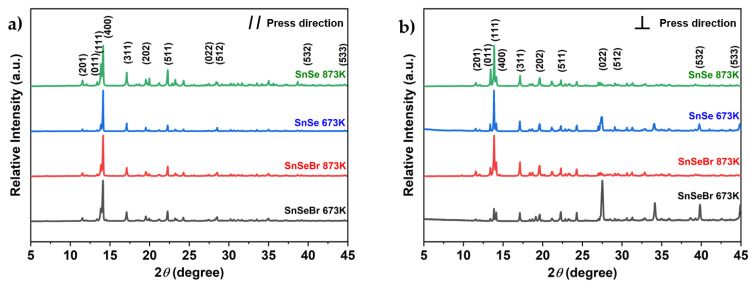
Room-temperature XRD patterns for bulk-sample SnSe and Br-doped SnSe (SnSe_0.9_Br), sintered at different temperatures, taken in the plane (**a**) parallel to the SPS pressing direction and (**b**) perpendicular to the SPS pressing direction.

Powder XRD is performed to ensure that an orthorhombic *Pnma* phase is obtained during synthesis. The resulting X-ray pattern shown in Figure 2 (full pattern in the Supplementary Materials, Figure S2) matches those reported in the literature [33], indicating that the chosen synthesis route was successful. Since an anisotropic ordering of the material was expected due to the use of SPS, the obtained samples had to be examined in different sample orientations. The reflection patterns show differences in intensities, indicating a microstructural anisotropy (Figure 3). The obtained samples were labelled according to their chemical composition and sintering temperatures. Undoped p-type SnSe samples were labelled “SnSe”, and n-type SnSe_0.9_Br_0.1_-based samples were called “SnSeBr”. The temperature stated afterwards refers to the sintering temperature in the SPS.

**Figure 4:**
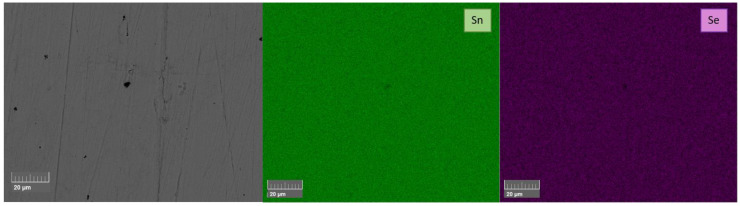
BSE image and corresponding EDX elemental mapping of undoped SnSe that was sintered by SPS at 773 K (SnSe 773 K).

**Figure 5:**
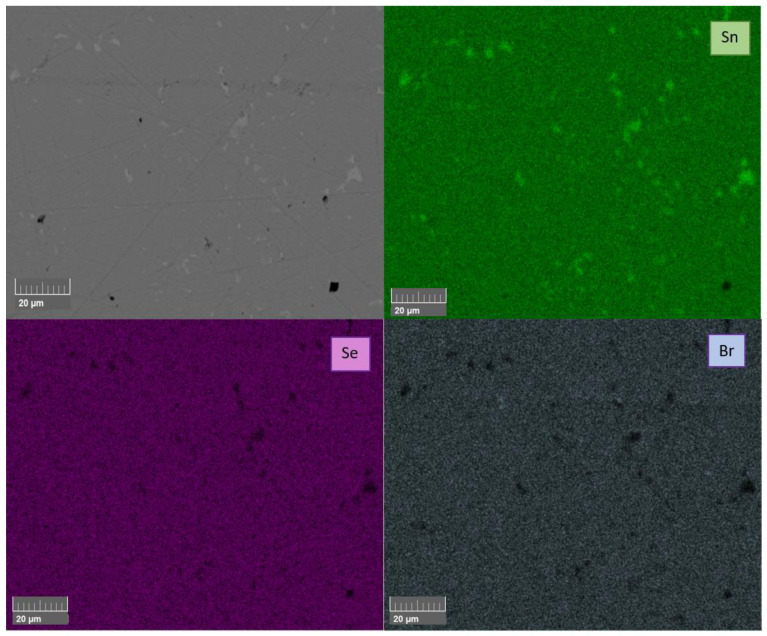
BSE image and corresponding EDX elemental mapping of Br-doped SnSe (SnSe_0.9_Br) that was sintered by SPS at 753 K (SnSeBr 753 K).

From the XRD patterns, it can be confirmed that a *Pnma* SnSe structure was obtained. However, all the SnSe_0.9_Br_0.1_-based samples showed a metallic tin secondary phase. All undoped SnSe samples did not show any secondary phase impurities in the XRD spectra. For further phase purity and elemental distribution analysis, SEM combined with EDX mapping was employed. Figure 4 and Figure 5 show the backscattered electron images and the elemental maps of the pure SnSe and Br-doped SnSe samples that were sintered at 753/773 K, just below the phase transition. For undoped SnSe, a uniform elemental distribution is shown with no visible phase segregation (Figure 4), whereas in Br-doped SnSe, pure tin secondary phases were found (Figure 5).

Table 1 summarises the actual chemical composition of all the prepared samples. The actual composition resulted from the average of three different point EDX scans. The results show that the actual composition deviated from the nominal composition in a lack of tin and thus an excess of Se. Moreover, the amount of Br found in the samples was less than expected. The shown lack of Sn can lead to improved TE properties, as was shown by Wei et al. [18].

Based on the different densities depending on the sintering temperature, sintering at *T* = 573 K resulted in a lower sample density compared to samples sintered at higher temperatures for the undoped samples. With sintering at a temperature ~200 K lower than the phase transition temperature, this behaviour of incomplete compaction is to be expected. However, the large difference in the density of single-crystalline and polycrystalline SnSe [13] cannot be entirely explained by different sintering temperatures. For Br-doped samples, the influence of sintering temperature was not observable. Due to the presence of a Sn secondary phase and the sintering temperature exceeding the melting point of Sn (*T* = 505 K), liquid Sn could fill out voids, resulting in densities independent of the sintering temperature in the SnSe_0.9_Br_0.1_-based samples.

### 3.2. Thermoelectric Properties

**Figure 6:**
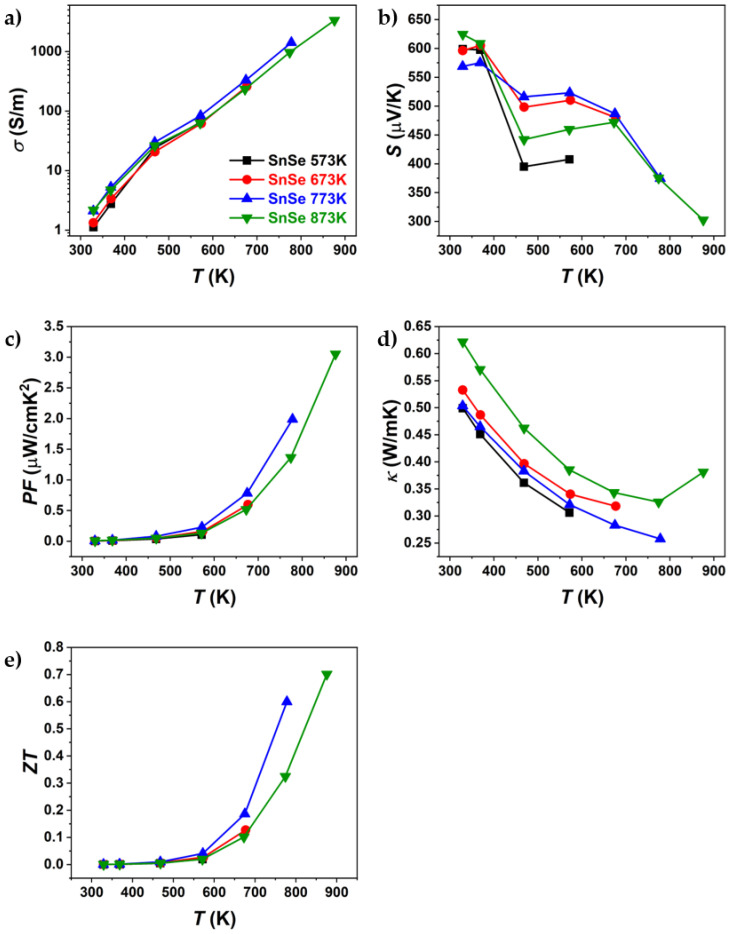
Temperature dependence of (**a**) electrical conductivity, (**b**) Seebeck coefficient, (**c**) power factor, (**d**) thermal conductivity, and (**e**) ZT of pure SnSe sintered at different temperatures. Only the first heating part of the heating–cooling measurements is shown.

**Figure 7:**
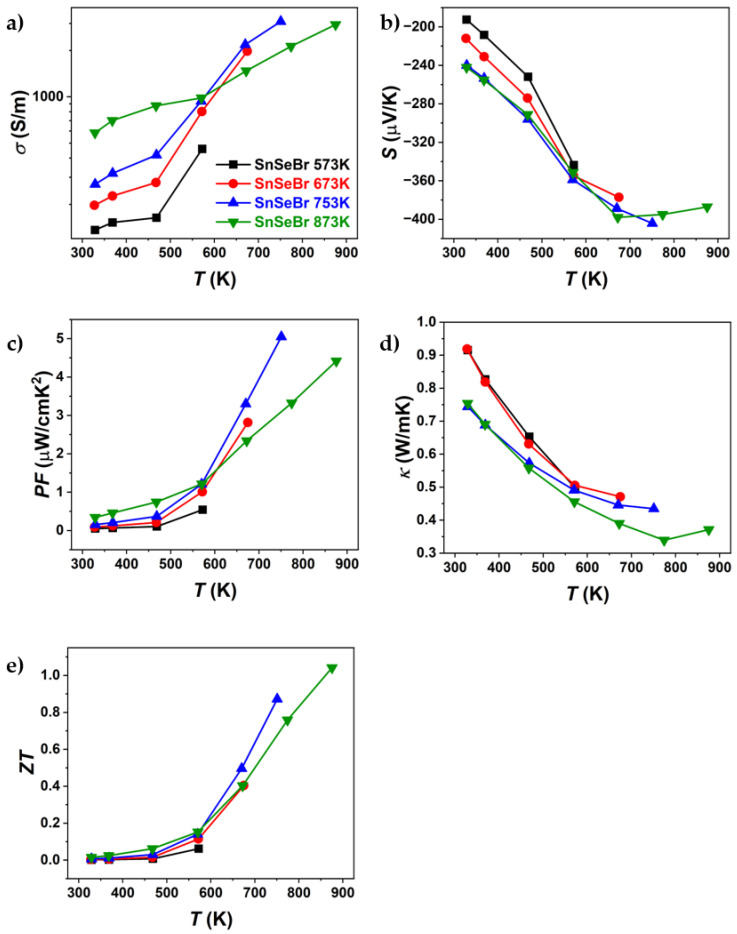
Temperature dependence of (**a**) electrical conductivity, (**b**) Seebeck coefficient, (**c**) power factor, (**d**) thermal conductivity, and (**e**) *ZT* of Br-doped SnSe (SnSe_0.9_Br), sintered at different temperatures. Only the first heating part of the heating–cooling measurements is shown.

The results of the thermoelectric property characterisation (electrical conductivity, Seebeck coefficient, thermal conductivity, power factor, and *ZT*) are shown in Figure 6 and Figure 7. The sample properties were measured parallel to the pressing direction, since it was reported that the highest TE performance can be obtained in that direction [12,35], as XRD results have shown anisotropic sample behaviour. TE properties were measured under heating and cooling. This was performed to observe if the values were repeatable or if a hysteresis was observed, as was reported by Chen et al. [36]. All compounds were only measured up to their sintering temperature to avoid potential changes in the sample. The results are shown only, as the first heating curves since the cooling TE properties did not match the heating measurements (Supplementary Materials, Figures S3 and S4).

Figure 6 and Figure 7 present the thermoelectric properties of Br-doped and pure SnSe. For all samples, an increase in electrical conductivity was observed (Figure 6a and Figure 7a), indicating semiconducting behaviour. After sintering at different temperatures, significant differences in electrical conductivity were observed, particularly for Br-doped samples. The most notable variations were seen in thermal conductivity, where, for Br-doped samples, *κ* decreased with increasing sintering temperature, reaching a minimum of ~0.34 W/mK at 773 K (SnSeBr 873 K). In contrast, pure SnSe showed the lowest thermal conductivity (~0.25 W/mK) for the sample sintered at 773 K. The Seebeck coefficient (Figure 6b and Figure 7b) was also dependent on the sintering temperature, with samples sintered at 873 K showing higher absolute values, while those sintered below the phase transition (753 K and 773 K) exhibited nearly identical values. The maximum *ZT* for the sample sintered at 753 K is comparable to values in the literature for similarly doped SnSe [35]. However, the highest *ZT* values were found at 873 K (1.04 for Br-doped and 0.7 for pure SnSe). Despite similar *ZT* values, the thermal conductivity reported in this work is significantly higher than the reported ~0.32 W/mK at 773 K [35], which could be attributed to tin secondary phases, contributing to the higher electrical conductivity and power factor. Potentially, higher *ZT* values might be achievable in samples sintered just below the phase transition, as they exhibited superior thermoelectric properties up to the highest measurement temperature. This raises the question of whether measurements above the phase transition should be considered, as structural or compositional changes could occur, affecting the replication of high *ZT* values. To evaluate potential property changes with temperature, TE properties were measured in three heating–cooling cycles up to 573 K, 673 K, 773 K, and 873 K. The samples used for the thermal cycling experiments were not previously measured.

**Figure 8:**
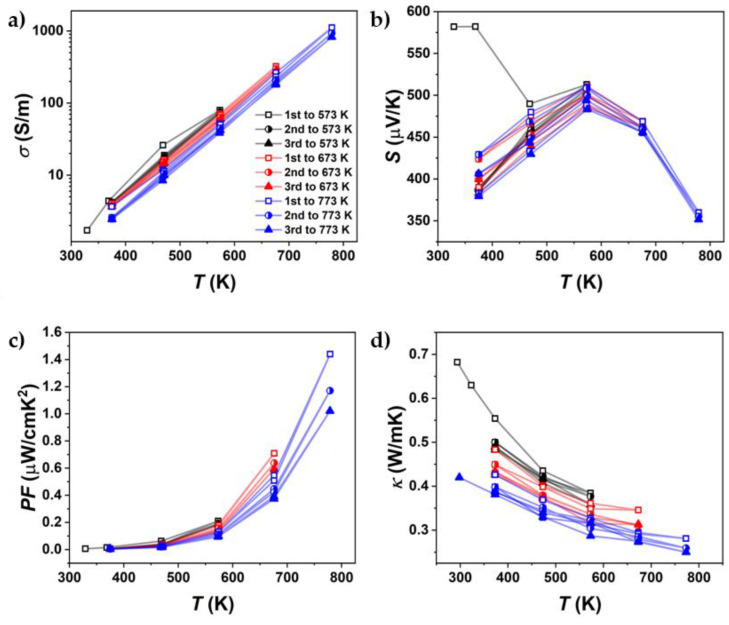
Cycling measurements up to different maximum temperatures of (**a**) electrical conductivity, (**b**) Seebeck coefficient, (**c**) power factor, and (**d**) thermal conductivity of undoped SnSe, sintered at 773 K (SnSe 773 K).

Figure 8 shows the results for one of these heating–cooling measurements with different peak temperatures (Supplementary Materials, Figures S5–S7). Differences between each cycle already occurred in the cycles with a peak temperature of 573 K. However, these changes mainly occurred in thermal conductivity. In the cycles up to 673 K, a larger decrease in both electrical conductivity and thermal conductivity was observed. This decrease was further amplified by the heating cycles up to 773 K. The change in TE properties was observed more significantly in Br-doped SnSe, but it also affected pure SnSe. Changes in electrical conductivity at high temperatures are less apparent due to the logarithmic scale used to highlight variations at lower temperatures. However, given the ~8 µV/K decrease in the Seebeck coefficient at 773 K, the power factor serves as a useful proxy for assessing electrical conductivity on a linear scale.

**Figure 9:**
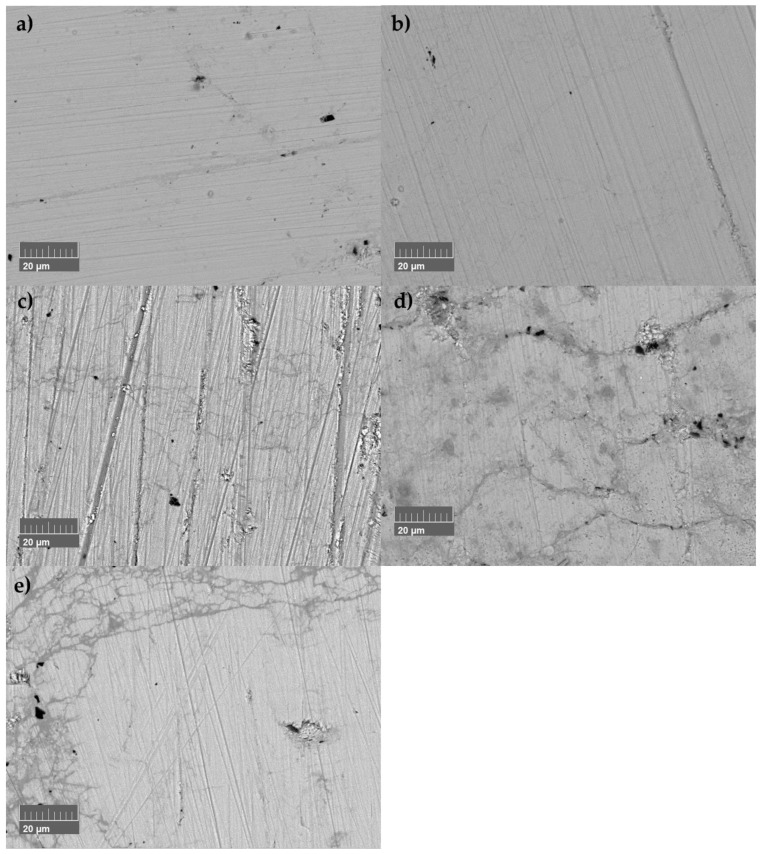
Microstructural evolution of undoped SnSe sample sintered at 873 K (SnSe 873 K) after several heating–cooling measurement cycles: (**a**) after SPS, (**b**) after measuring three times up to 573 K, (**c**) after measuring three times up to 673 K, (**d**) after measuring three times up to 773 K, and (**e**) after measuring one time up to 873 K.

Since the instabilities in thermoelectric properties occur for both undoped SnSe and SnSe_0.9_Br_0.1_-based samples, the unrepeatable transport properties measured within the heating and cooling cycles must be independent of the occurrence of Sn secondary phase impurities or Br-doping. Based on the amplitude of these decreases, local inhomogeneities induced by SnBr_2_-doping accelerate the phenomenon. To exclude surface effects, a sample (pure SnSe sintered at 873 K) was polished after each heating–cooling measurement cycle and its microstructure and composition were examined by SEM to document possible microstructural and compositional evolutions (Figure 9, corresponding to TE properties in Figure S8).

It has to be considered that the recorded SEM images were not always taken at the same spot. However, microstructure evolution is observable, the amount of microcracks and their size increasing after the corresponding thermal cycling, with large numbers of cracks and damage to the microstructure after passing the phase transition once (Figure 9e). The sample suffered a mechanical failure during polishing for further measurements due to the damaged microstructure. The presence of a damaged microstructure can explain changes in electrical conductivity and thermal conductivity due to the decrease in the mean free path for phonons and electrons. Cracks do not compromise the Seebeck coefficient, and this is evidenced by the Seebeck measurements. Since smaller changes in the Seebeck coefficient were observed, the accompanying changes in chemical composition had to be discussed. Therefore, thermal analysis methods were utilised. Furthermore, all samples were subject to annealing at *T* = 753 K for two weeks. The expectation was that changes during heating and cooling would not occur after the annealing process if the process was temperature-induced.

### 3.3. Thermal Analysis Results

**Figure 10:**
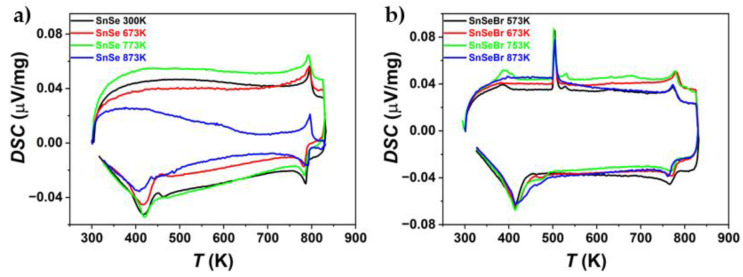
DSC measurements of (**a**) undoped SnSe and (**b**) Br-doped SnSe (SnSe_0.9_Br), sintered at different temperatures. The measurement shows the first heating and cooling cycle of the sample pieces.

**Figure 11:**
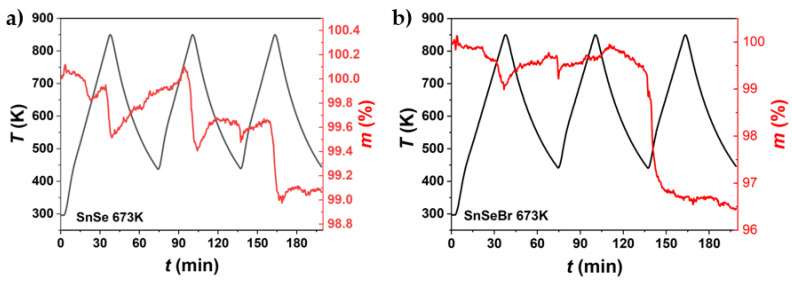
TGA measurement in three heating and cooling cycles of (**a**) undoped SnSe and (**b**) Br-doped (SnSe_0.9_Br), both sintered at 673 K. The samples were not subject to heating–cooling before the TGA measurement.

Since the TE property measurements indicated that the synthesised SnSe and Br-doped SnSe were not stable during thermal cycling, DSC and TGA were employed to understand the progressive decomposition during heating and cooling cycles (Figure 10 and Figure 11). For Br-doped samples, the presence of a Sn secondary phase is confirmed by the melting peak of Sn at ~*T* = 505 K. In two cases, a second peak follows at ~*T* = 529 K. Since no melting for any of the components or combination of components occurs at this temperature, it is considered another thermal reaction or an artefact of the DSC measurement, as it was not repeatably found. The phase transition from *Pnma* to the *Cmcm* phase can be seen for all samples, as well as the corresponding peak when cooling. For Br-doped samples, the phase transition starts at about *T* = 760 K, with a peak at ~*T* = 777 K. For pure SnSe, it starts at ~*T* = 775 K, with a peak at *T* = 788 K. Doping SnSe with Br lowers the phase transition temperature.

TGA was employed to observe mass changes during thermal cycling. For pure SnSe, mass changes occurred at about *T* = 813 K. For Br-doped samples, an additional mass change occurred at about *T* = 653 K. Slight continual increases in mass were observed due to oxidation during the measurement. The common loss in weight at *T* = 813 K can be attributed to the evaporation of selenium. This is consistent with the observation during electrical conductivity and Seebeck measurement, where Se evaporation was present in measurements, with peak temperatures above the phase transition (Supplementary Materials, Figure S9). TGA measurements were also not completely reproducible/repeatable, as mass gains and losses occurred at arbitrary points in time during the thermal cycling. The only consistency was that weight losses happened at about *T* > 813 K. For undoped SnSe, changes in chemical composition are unlikely to be the cause of the degradation of thermoelectric properties, as mass changes mainly occur above the phase transition temperature, while instabilities in thermoelectric properties already exist at temperatures far below the phase transition temperature.

Furthermore, a small mass decrease was observed at *T* = 565 K during the first cycle. This could be attributed to the sublimation of SeO_2_. Since the TGA measurement could only be performed under Ar-flushing conditions, oxidation during the measurement cannot be ruled out entirely.

### 3.4. The Effects of Annealing

The fresh-made SnSe and Br-doped samples were all sealed in a quartz tube under an argon atmosphere and were kept at a temperature below the phase transition temperature, which DSC determined to start at ~*T* > 760 K for Br-doped SnSe. Afterwards, the same measurement procedures that were used for the unannealed sample were repeated. The samples used for annealing were not subjected to previous heat treatments/measurements.

**Figure 12:**
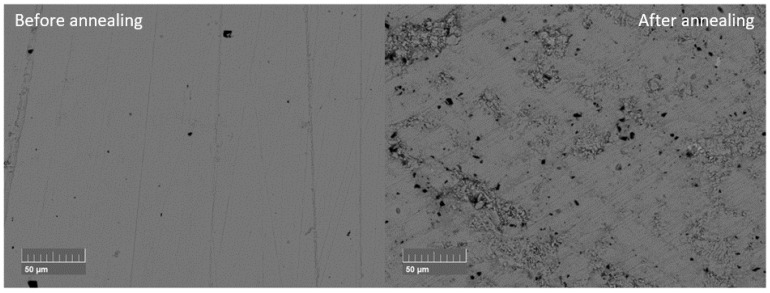
BSE images before and after annealing of undoped SnSe sintered at 773 K (SnSe 773 K).

After annealing, all samples still showed the *Pnma* phase (Supplementary Materials, Figure S10). The Sn secondary phase peak disappeared from the XRD pattern. In the EDX measurements, small Sn secondary phases can be determined. During annealing, Sn beads formed on the sample surface of the SnSe_0.9_Br_0.1_-based samples, explaining the reduction in the Sn secondary phase in the matrix (Supplementary Materials, Figure S11). Changes in the main phase composition were not observed. This confirms the previous thermal analysis results, where no evaporation losses were anticipated, at least in undoped SnSe. Additionally, the density decreased for all samples after annealing between 1 and 10% (Supplementary Materials, Table S1). The changes in density were likely due to the increased damage to the microstructure. The damage to the microstructure was also observed in a comparison of BSE images of the same sample (Figure 12) recorded before and after the annealing process. Moreover, annealed samples were more brittle than before, resulting in easier breaking during polishing.

**Figure 13:**
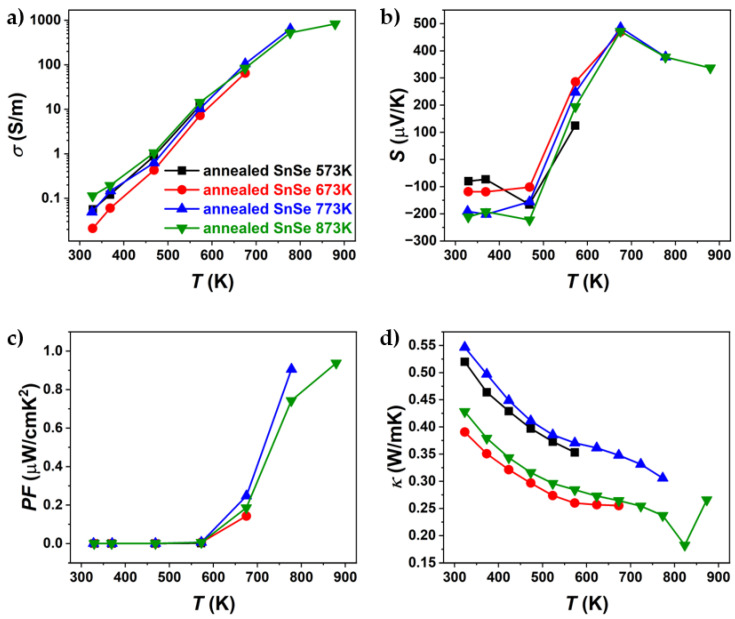
Temperature dependence of (**a**) electrical conductivity, (**b**) Seebeck coefficient, (**c**) power factor, and (**d**) thermal conductivity of pure SnSe, sintered at different temperatures, where all samples were annealed at 753 K.

**Figure 14:**
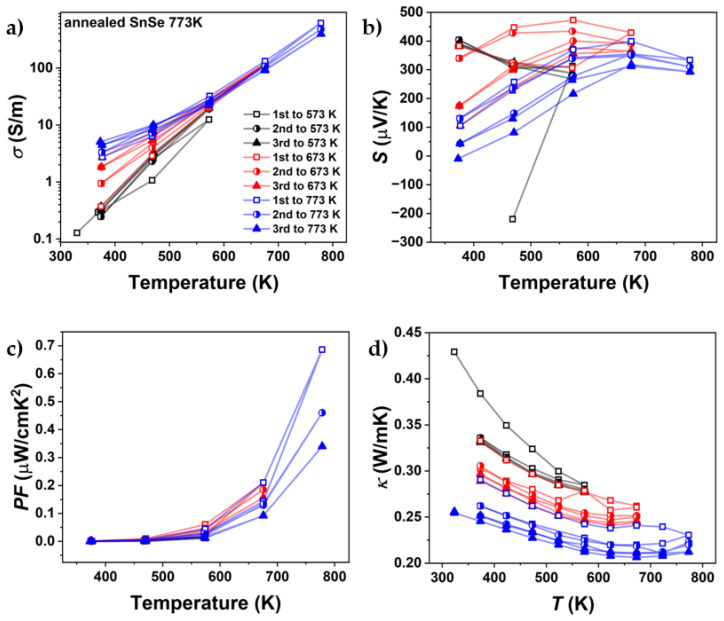
Cycling measurements up to different maximum temperatures of (**a**) electrical conductivity, (**b**) Seebeck coefficient, (**c**) power factor, and (**d**) thermal conductivity of undoped SnSe, sintered at 773 K (SnSe 773 K), that was annealed for two weeks.

Figure 13 illustrates the results of thermoelectric property measurements for pure SnSe after two weeks of annealing (SEM data after annealing in Supplementary Materials, Figures S12 and S13). Compared to the measurements taken before annealing, the power factor dropped from 1.99 µW/mK to 0.99 µW/mK. This is mainly attributed to the decrease in electrical conductivity, which declined by about 75%. While electrical conductivity significantly decreased, the Seebeck coefficient reached the same values at higher temperatures (*T* > 673 K) as in the measurements prior to annealing. During the first cycle for undoped SnSe, the Seebeck coefficient was negative, a phenomenon only noted after the first measurement cycle. After the first cycle, the Seebeck coefficient remained positive (Figure 14b). Thermal conductivity also decreased as a result of annealing, lower than that of single-crystalline SnSe. These findings of the first measurement after annealing align with the observation of an increased number and growth of microcracks. Since the annealing was conducted with the expectation of either chemical and/or structural changes, cycling measurements were performed again to observe whether thermoelectric properties changed after excessive annealing.

Figure 14 presents the heating–cooling cycle transport properties of the annealed SnSe 773 K sample. Prior to annealing, the samples exhibited instability across all measured thermoelectric properties, indicating that the system had not yet reached equilibrium, the primary goal of the annealing process. This aligns with the hypothesis that crack formation and microstructural defects contribute significantly to the instability observed in the SnSe system. Furthermore, the Seebeck coefficient exhibited notable changes following annealing. While prior measurements showed consistent values at temperatures ≥ 673 K (Figure 8b), a post-annealing measurement revealed an ~13% reduction at 773 K. This could indicate further changes in chemical composition during the measurement, which, in addition to the damage to the microstructure, can contribute to the degradation of thermoelectric properties.

These findings suggest that SnSe compounds produced using the method described in this work are not suitable for long-term applications, as they suffer from both declining thermoelectric performance and mechanical instability. Prolonged annealing below the phase transition temperature did not enhance the thermoelectric properties; instead, it appeared to accelerate their degradation.

To conclude whether this behaviour was related to the chosen experimental methods or intrinsic material properties of SnSe, a literature search was conducted, including a variety of papers, to find differences in synthesis, application temperature, and, if possible, stability data in heating–cooling (Table 2, complete table in Supplementary Materials). It showed that a variety of polycrystalline SnSe synthesis methods exist that are mainly different in their temperature programs. Many differences lie in the cooling programs or intermediate steps in opposition to a simple furnace cooling to room temperature [35,37,38,39], quenching [36,40,41,42], or annealing after the first melting step [12,40,43,44]. Moreover, it is also evident that for some works, as in this work, the highest *ZT* was measured at temperatures above the phase transition [16,39,40,42,45,46,47,48,49,50,51,52,53,54,55,56]. This could be problematic in terms of mechanical stability and performance degradation. Therefore, stability tests of SnSe compounds should be included when discussing the usability of the presented compound for a thermoelectric device. Otherwise, proposed unreproducible high *ZT* values might not be trustworthy for long-term application unless at least an uncertainty quantification is applied.

Similar observations of SnSe instabilities were also reported by Lo et al. [57], who found that microcracks formed after SPS and expanded further during thermal cycling. Since the cause of the reduction in TE performance was validated, the cause of the appearance of the microcracks has to be clarified. One mechanism for crack formation in SnSe materials is the phase transition from the high-temperature *Cmcm* phase to the room-temperature *Pnma* phase. This transformation is accompanied by an expansion that can generate large stresses, potentially breaking quartz tubes and causing cracks along the bc-plane in single-crystalline SnSe [32]. Since instabilities occurred in samples sintered at temperatures far below the phase transition temperature, only the starting material underwent a phase transition during the melting procedure. However, since ball milling was utilised, the defects introduced by ball milling were likely to be larger than those inflicted by the phase transition. Additionally, a reorientation of the grains during heating and cooling can be considered as a driving force for the degradation in the microstructure. Overall, it would be necessary to find a synthesis route for polycrystalline SnSe that can ensure a stable product. This has already been shown in several studies, where heating–cooling cycles were recorded with no significant difference in heating and cooling behaviour [15,36,45,47,54,58]. Long-term tests have to be considered regardless of single heating–cooling tests.

**Table 2:** Table of different solid-state synthesis methods in the literature.

	Synthesis Parameter			Intermediate Step		Measurement				
Dopants	*T* (K)	*t* (h)	Cooling	*T* (K)	*t* (h)	*T*_max_ (K)	*T_ZT_*_max_ (K)	*ZT*_max_	Heating / cooling	Ref.
Br	1223	10	-	-	-	873	873	1.04	yes	This work
Er, Ho, Sm, Pr, Yb	1223	8	-	-	-	900	873	2.1	no	[16]
Te	1223	24	Down to 1123 K in 0.2 K/min	-	-	900	875	1.6	no	[56]
Na	1193	1	-	-	-	830	780	0.9	no	[23]
Na, Pb	1223	12	-	613	6 h under Ar/H_2_, after ball milling	773	773	2.5	no	[17]
Pb, Br	1223	6	-	-	-	773	773	1.2	no	[59]
CeCl_3_	1273	1	-	-	-	773	773	1.17	no	[60]
Na	1223	12	-	773	48 h, 613 K, 6 h, Ar/H_2_	783	783	3.1	no	[12]
Ag	1200	12	-	800	72 h	750	750	0.6	yes	[36]
S, I	1193	6	To 873 K in 100 K/h, kept for 70 h at 873 K	-	-	773	773	1	yes	[61]
MoCl_5_	1223	10	-	-	-	798	798	2	yes	[15]

## 4. Conclusions and Outlook

Our investigation focused on the impact of different sintering temperatures on the thermoelectric properties of p-type SnSe and n-type SnSe_0.9_Br_0.1_-based materials. The highest *ZT* values were 1.04 for the SnSe_0.9_Br_0.1_-based material and 0.7 for undoped SnSe, both at *T* = 873 K. However, during heating and cooling cycles, all materials exhibited a decrease in thermoelectric performance, attributed to the growth and formation of microcracks. Further investigation shows that the activation of these dynamic processes depends on the temperature used, with the amplitude of these changes increasing as the temperature rises. It was demonstrated that annealing at a temperature below the phase transition temperature influences thermoelectric performance. Post-annealing, all thermoelectric properties degraded, with the highest *ZT* in the Br-doped material being *ZT* = 0.49 and that in undoped SnSe being *ZT* = 0.31 at *T* = 873 K. Moreover, the degradation of the microstructure during thermal cycling could not be mitigated by the extended annealing process.

This study highlights the importance of comprehensive and reproducible data regarding sample stability, going beyond the presentation of a single exceptional *ZT* value. While our initial goal of establishing a clear correlation between sintering temperatures above the phase transition and low thermal conductivities may not have been achieved, our results do illuminate the broader issue of stability in SnSe compounds, which is likely a consequence of experimental methodologies and resulting ion/effect dynamics, kinetics, and thermodynamics. Therefore, there is a pressing need for a deeper understanding of the reasons behind the observed degradation, as well as the factors responsible for its absence in other scenarios. Going forward, it will be essential to include both heating and cooling measurements for SnSe compounds when reporting high *ZT* values, as stability must be a key consideration if SnSe is to be a viable candidate for future thermoelectric applications.

## Figures and Tables

**Table 1:** Measured average phase composition according to EDX point analysis and density after SPS sintering at different temperatures. The measurement error bars for the elements are ±2% for Sn, ±4% for Se, and ±15% for Br.

Sample Name	Real Composition	Density (g/cm^3^)
SnSe 573 K	Sn_0.92(6)_Se_1.07(4)_	6.01 (0)
SnSe 673 K	Sn_0.91(9)_Se_1.08(1)_	6.11 (8)
SnSe 773 K	Sn_0.92(3)_Se_1_._07(7)_	6.11 (5)
SnSe 873 K	Sn_0.92(1)_Se_1.07(9)_	6.08 (9)
SnSeBr 573 K	Sn_0.91(9)_Se_1.04(2)_Br_0.03(9)_	6.08 (5)
SnSeBr 673 K	Sn_0.93(0)_Se_1.02(8)_Br_0.04(2)_	6.05 (3)
SnSeBr 753 K	Sn_0.93(1)_Se_1.02(2)_Br_0.04(7)_	6.08 (3)
SnSeBr 873 K	Sn_0.91(3)_Se_1.05(4)_Br_0.03(2)_	6.07 (7)

## Data Availability

The original contributions presented in this study are included in the article/Supplementary Material. Further inquiries can be directed to the corresponding author.

## Supplementary Materials

The following supporting information can be downloaded at: https://www.mdpi.com/article/10.3390/ma18184228/s1, Figure S1. Sketch of the sample dimensions after SPS and subsequent cutting and the measurement of the thermal and electrical transport properties in parallel direction to the SPS uniaxial pressure direction. Figure S2. Powder XRD patterns of SnSe0.9Br0.1-based samples (“Br”) and pure SnSe (SnSe), sintered at different SPS temperatures (number in Kelvin). Figure S3. First heating-cooling measurement of (a) electrical conductivity, (b) Seebeck coefficient and (c) Power factor of SnSe0.9Br0.1-based materials sintered at different SPS temperatures. Figure S4. First heating-cooling measurement of (a) electrical conductivity, (b) Seebeck coefficient and (c) Power factor of pure SnSe materials sintered at different SPS temperatures. Figure S5. Cycling measurements up to different maximum temperatures of (a) electrical conductivity, (b) Seebeck coefficient and (c) thermal conductivity of SnSe0.9Br0.1-based material sintered at 753 K. Figure S6. Cycling measurements up to different maximum temperatures of (a) electrical conductivity, (b) Seebeck coefficient and (c) thermal conductivity of SnSe0.9Br0.1-based material sintered at 873 K. Figure S7. Cycling measurements up to different maximum temperatures of (a) electrical conductivity, (b) Seebeck coefficient and (c) thermal conductivity of pure SnSe material sintered at 873 K. Figure S8. Figure 9 Cycling measurements up to different maximum temperatures of (a) electrical conductivity and (b) Seebeck coefficient of pure SnSe material sintered at 873 K. This sample was polished after each heating-cooling cycle to avoid possible surface effects. Figure S9. ZEM-3 device after measurement of sample up to 873 K. Figure S10. Room temperature XRD patterns of powder sample SnSe and Br-doped (SnSe0.9Br0.1) SnSe with a literature comparison after two weeks of annealing. Figure S11 SnSe0.9Br0.1-based material after 336 h annealing at 753 K. Sn was found on the surface. Figure S12. BSE image and corresponding EDX elemental mapping of undoped SnSe that was sintered by SPS at 773 K (SnSe-773) after two weeks of annealing. Figure S13. BSE image and corresponding EDX elemental mapping of Br-doped SnSe that was sintered by SPS at 753 K (Br-753) after two weeks of annealing. Table S1. Actual phase composition and density after SPS sintering at different temperatures after two-week annealing. Figure S14. Temperature dependence of (a) electrical conductivity, (b) Seebeck coefficient, (c) Power factor and (d) thermal conductivity of Br-doped SnSe, sintered at different temperatures that were all annealed at 753 K.
