# Supplementary material for: Stability of SnSe-Based Thermoelectric Compounds
Source: Materials (Basel). 2025 Sep 9;18(18):4228. doi: 10.3390/ma18184228 (PMC12471590; doi:10.3390/ma18184228)
Supplement: Supplementary file 1 [file materials-18-04228-s001.zip › materials-3801060-supplementary.pdf]

Supporting Information

Stability of SnSe-based Thermoelectric Compounds

Moritz Thiem [a], Ann-Katrin Emmerich [a], Iliya Radulov [b], Anke Weidenkaff [a], Wenjie Xie [a,*]

a) Materials and Resources, Institute of Materials Science, Technical University of Darmstadt, Darmstadt 64287, Germany;

moritz.thiem@mr.tu-darmstadt.de (M.T); ann-katrin.emmerich@mr.tu-darmstadt.de (A.E); anke.weidenkaff@mr.tu-darmstadt.de (A.W)

b) Fraunhofer Research Institution for Materials Recycling and Resource Strategies IWKS, Hanau 63457, Germany

iliya.angelov.radulov@iwks.fraunhofer.de (I.R)

Corresponding email: wenjie.xie@mr.tu-darmstadt.de (W.X)

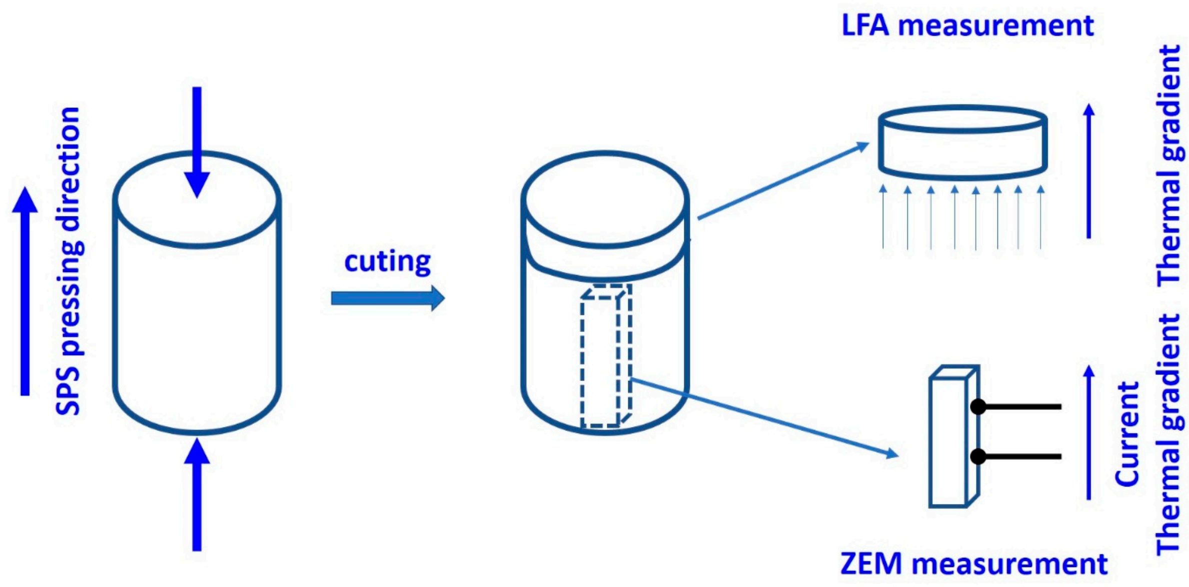

Figure S1 Sketch of the sample dimensions after SPS and subsequent cutting and the measurement of the thermal and electrical transport properties in parallel direction to the SPS uniaxial pressure direction.

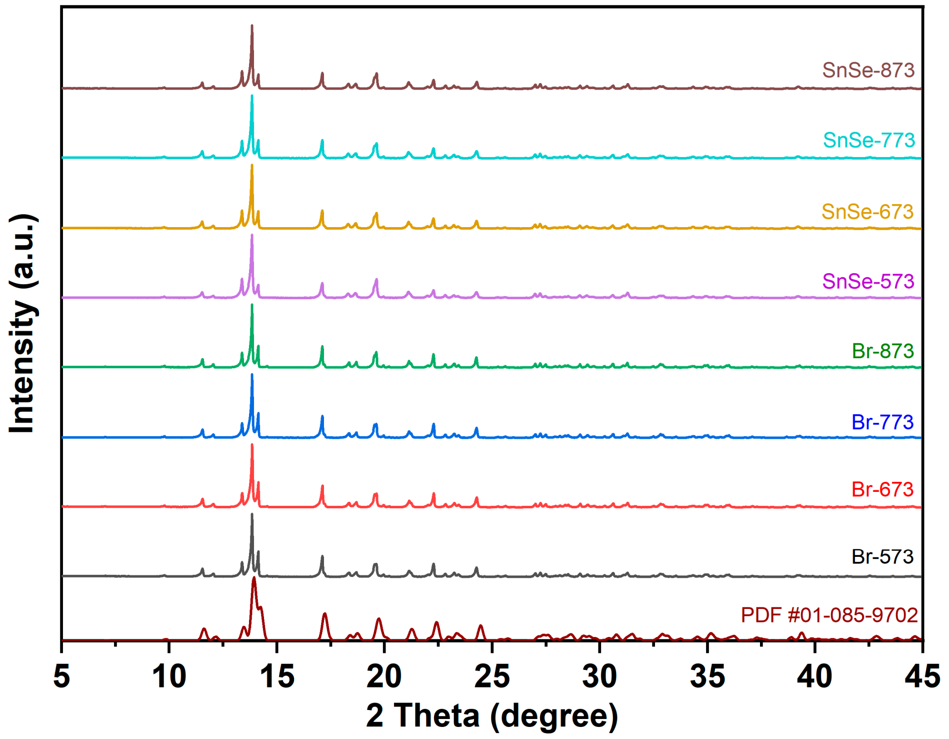

Figure S1 Powder XRD patterns of $SnSe_{0.9}Br_{0.1}$-based samples ("Br") and pure SnSe (SnSe), sintered at different SPS temperatures (number in Kelvin)

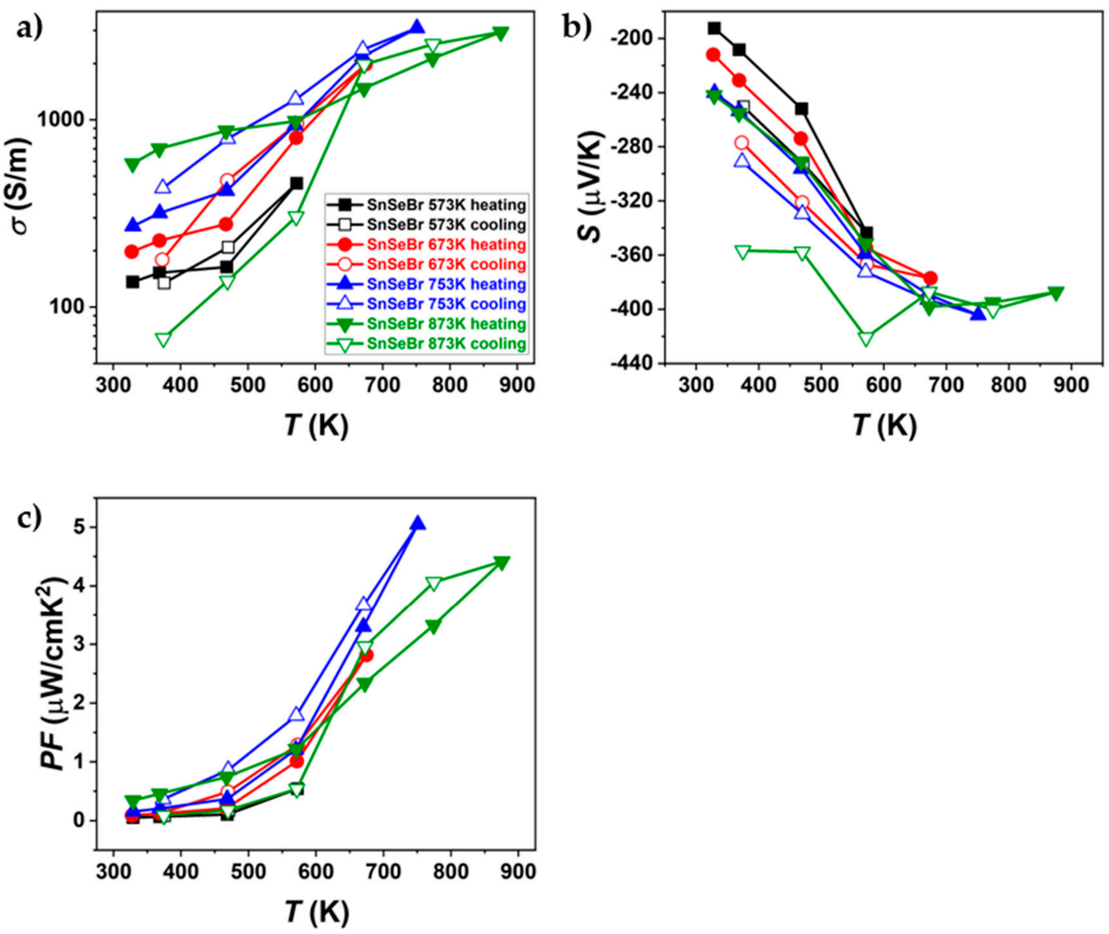

Figure S2 First heating-cooling measurement of a) electrical conductivity, b) Seebeck coefficient and c) Power factor of $SnSe_{0.9}Br_{0.1}$-based materials sintered at different SPS temperatures

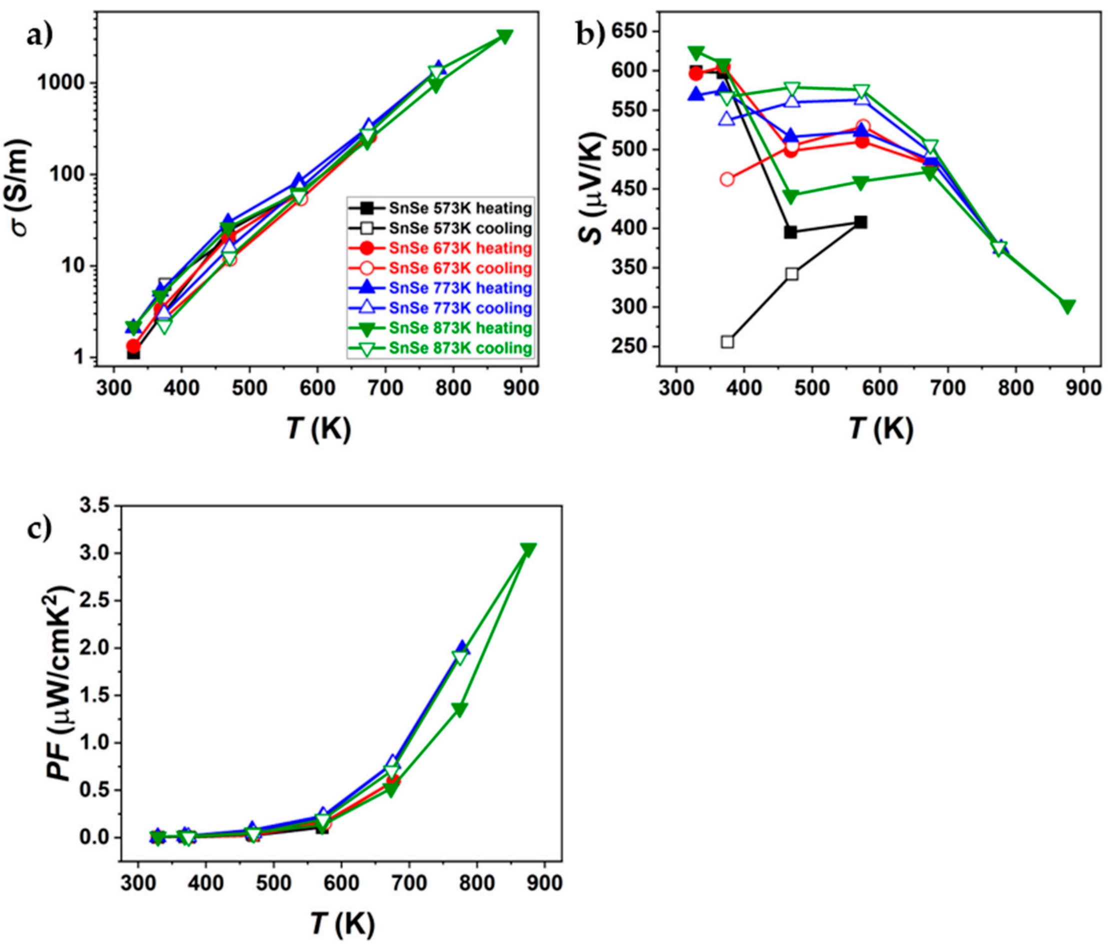

Figure S3 First heating-cooling measurement of a) electrical conductivity, b) Seebeck coefficient and c) Power factor of pure SnSe materials sintered at different SPS temperatures

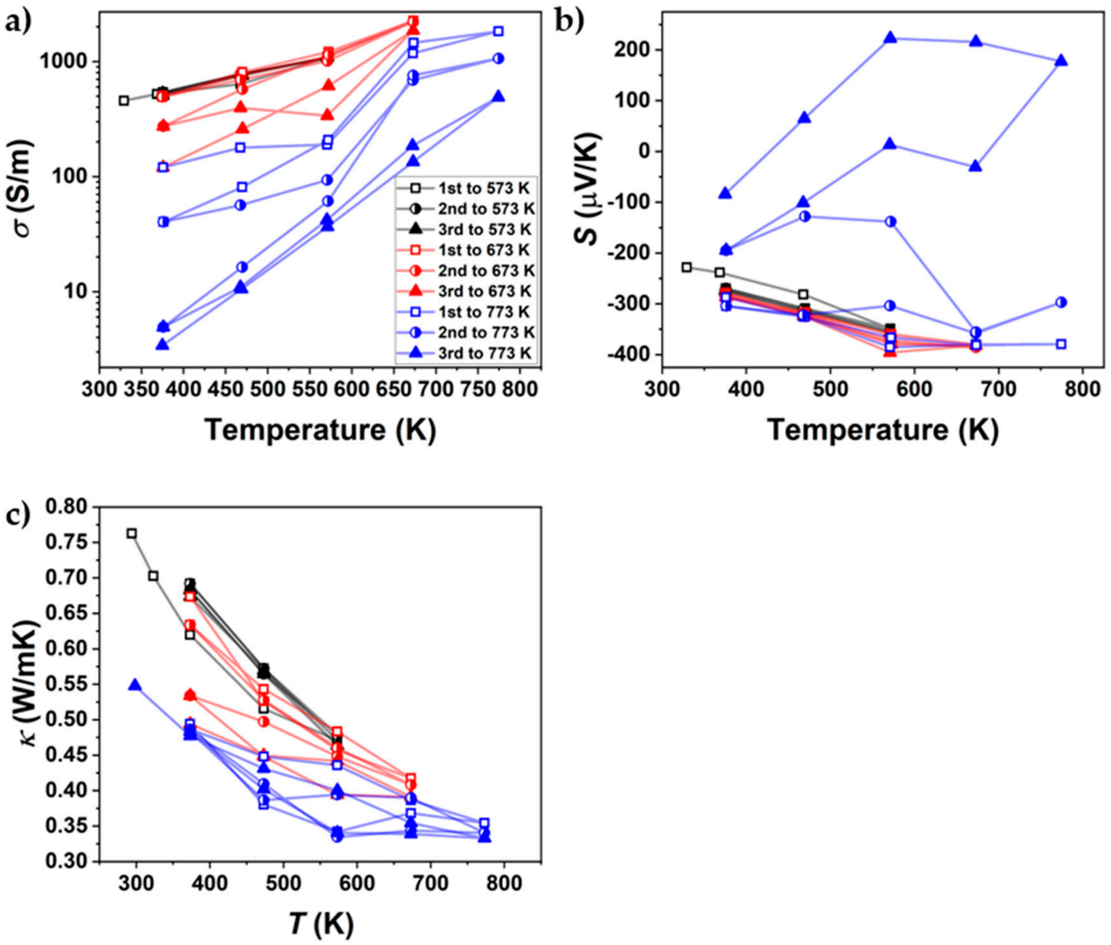

Figure S4 Cycling measurements up to different maximum temperatures of a) electrical conductivity, b) Seebeck coefficient and c) thermal conductivity of $SnSe_{0.9}Br_{0.1}$-based material sintered at 753 K

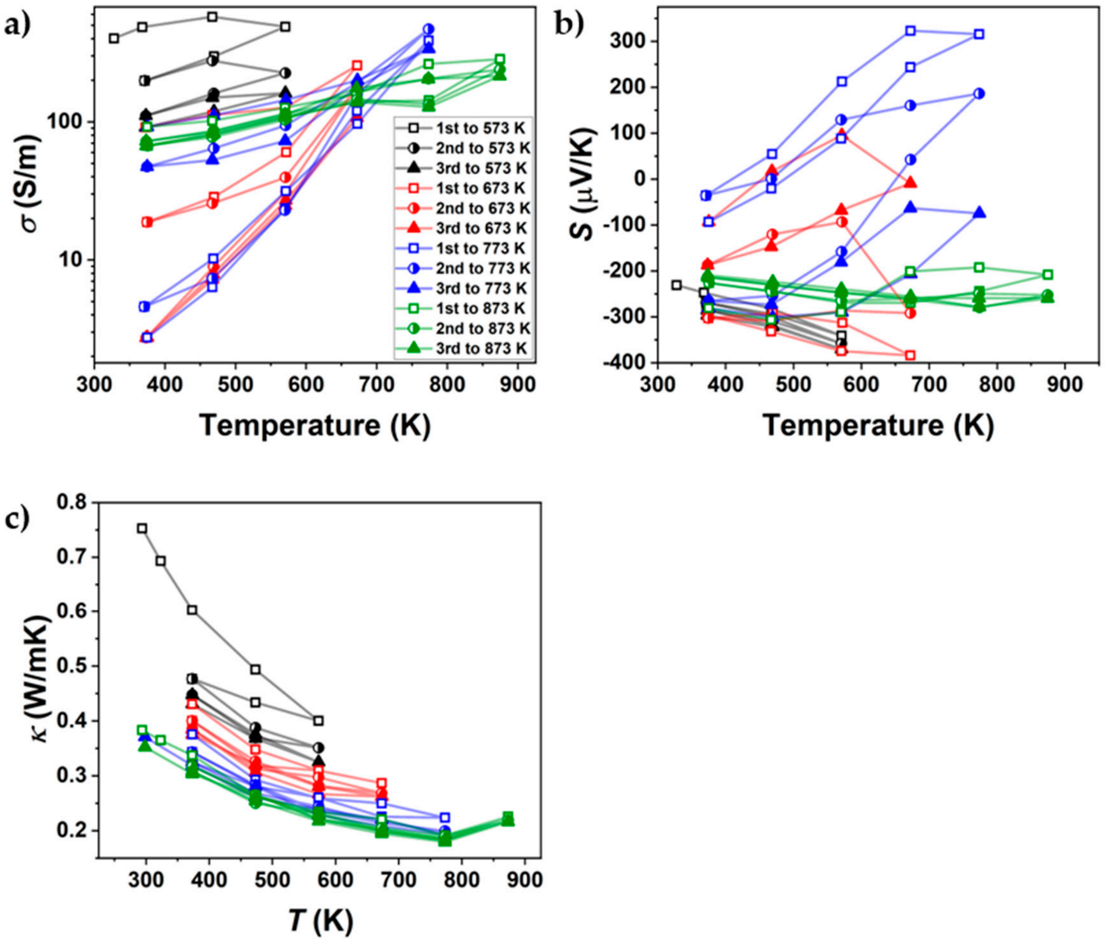

Figure S5 Cycling measurements up to different maximum temperatures of a) electrical conductivity, b) Seebeck coefficient and c) thermal conductivity of $SnSe_{0.9}Br_{0.1}$-based material sintered at 873 K

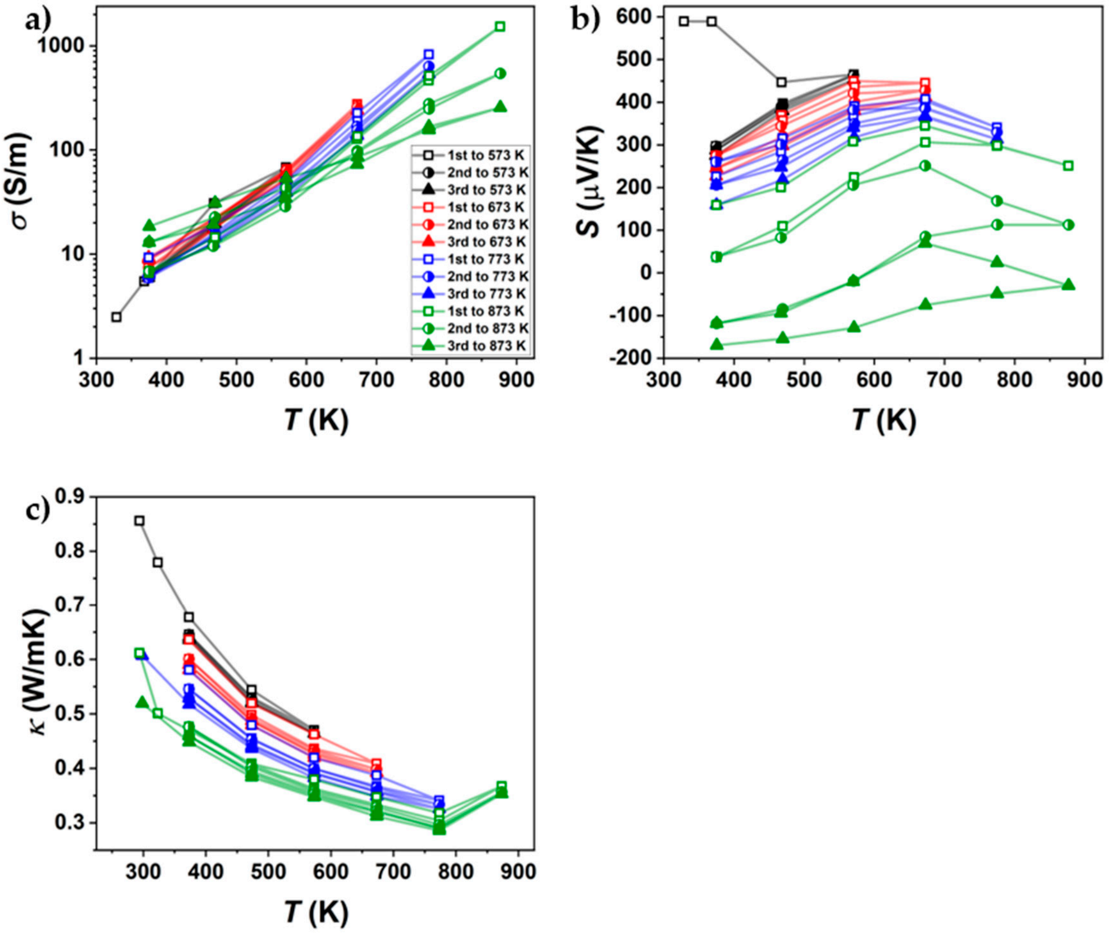

Figure S6 Cycling measurements up to different maximum temperatures of a) electrical conductivity, b) Seebeck coefficient and c) thermal conductivity of pure SnSe material sintered at 873 K.

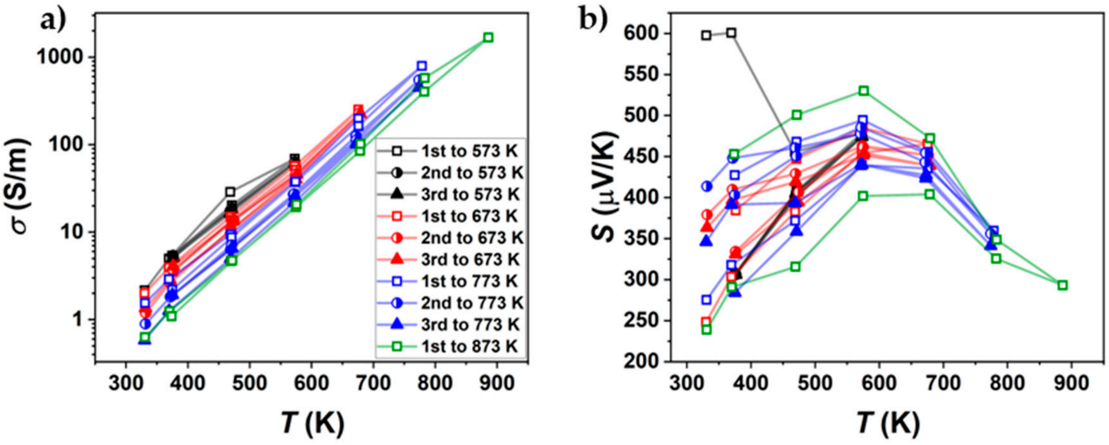

Figure S7 Figure 8 Cycling measurements up to different maximum temperatures of a) electrical conductivity and b) Seebeck coefficient of pure SnSe material sintered at 873 K. This sample was polished after each heating-cooling cycle to avoid possible surface effects.

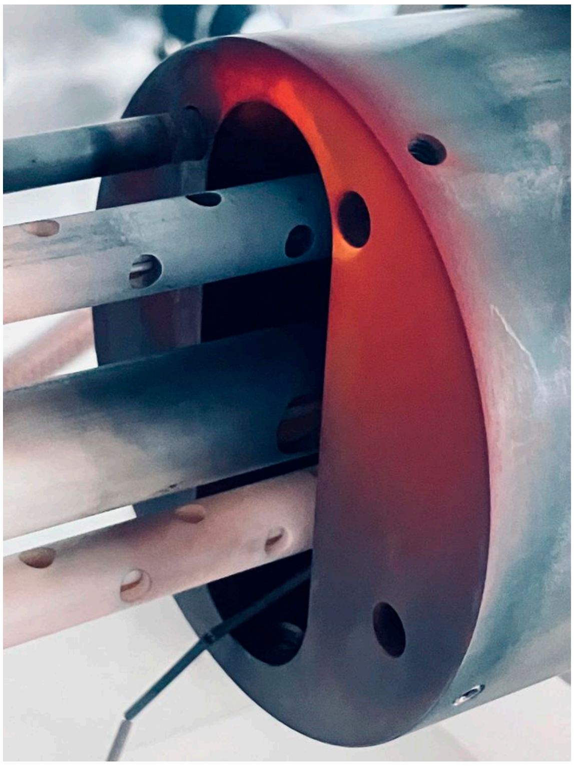

Figure S8 ZEM-3 device after measurement of sample up to 873 K.

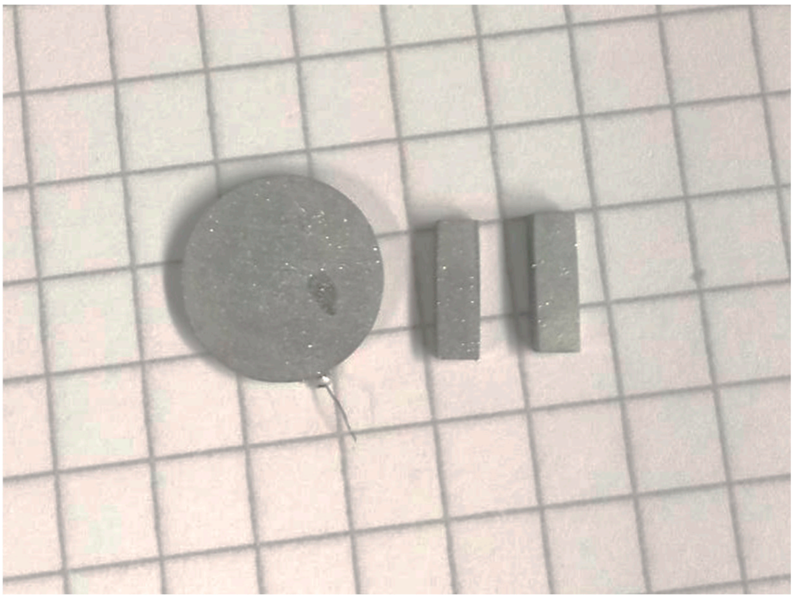

Figure S9 $SnSe_{0.9}Br_{0.1}$-based material after 336 h annealing at 753 K. Sn was found on the surface

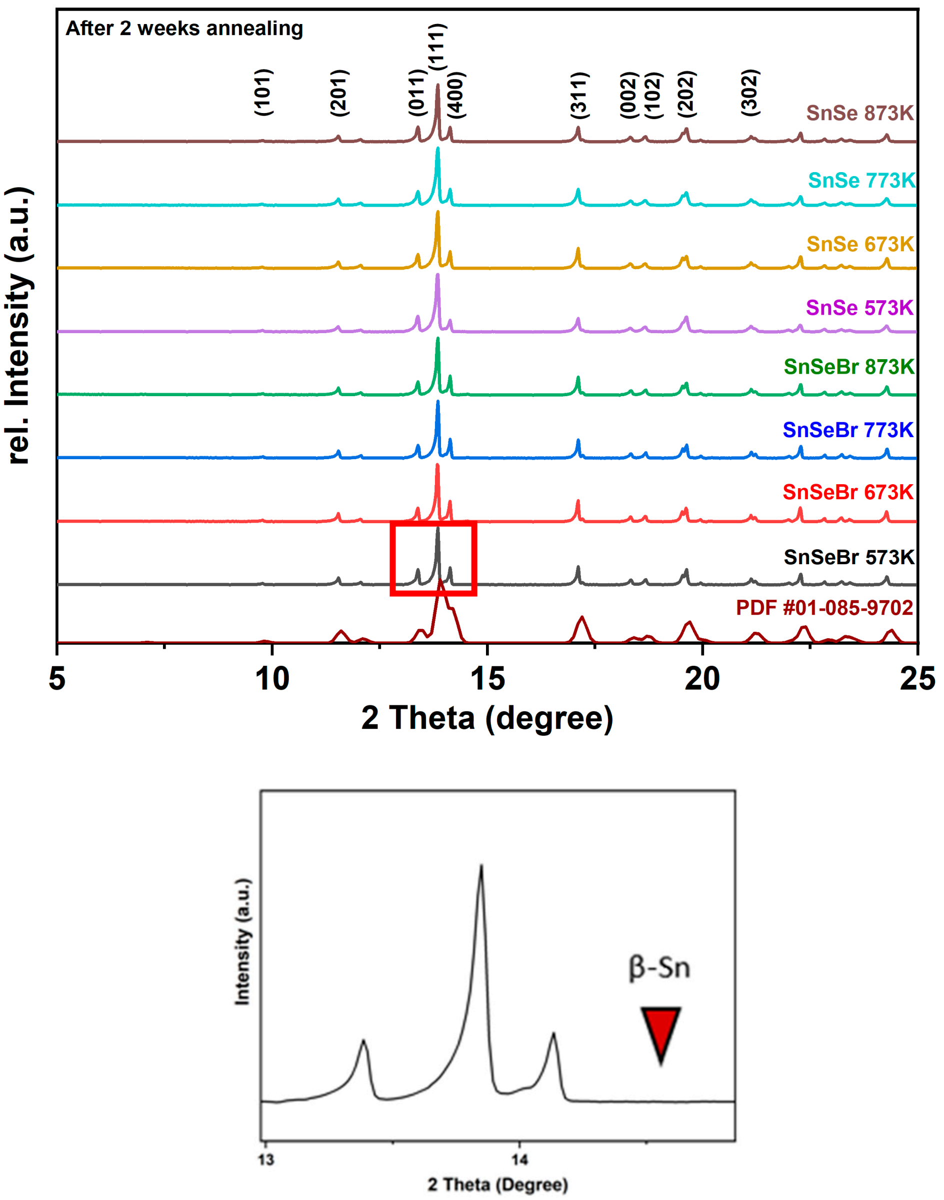

Figure S10 Room temperature XRD patterns of powder sample SnSe and Br-doped ($SnSe_{0.9}Br_{0.1}$) SnSe with a literature comparison [38] after two weeks of annealing.

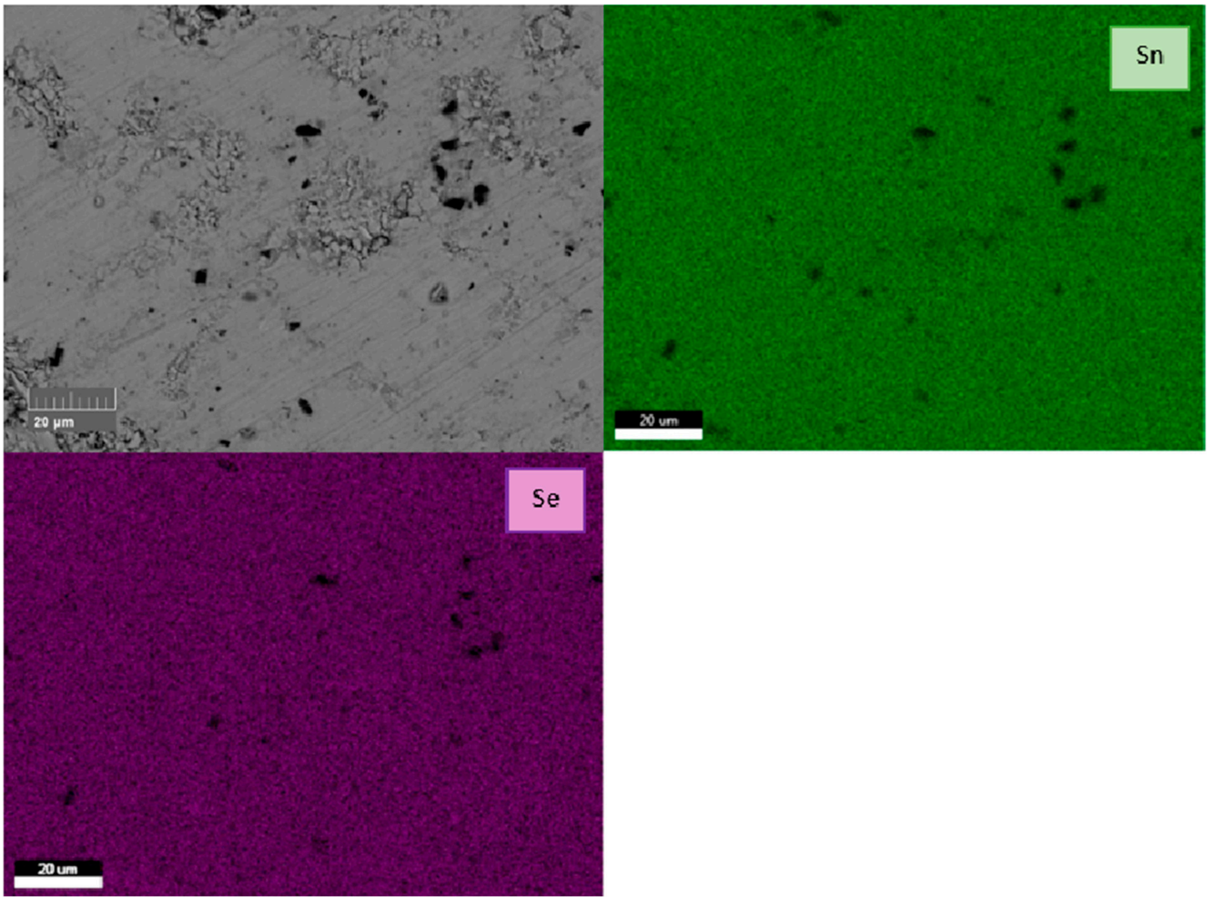

Figure S11 BSE image and corresponding EDX elemental mapping of undoped SnSe that was sintered by SPS at 773 K (SnSe-773) after two weeks of annealing

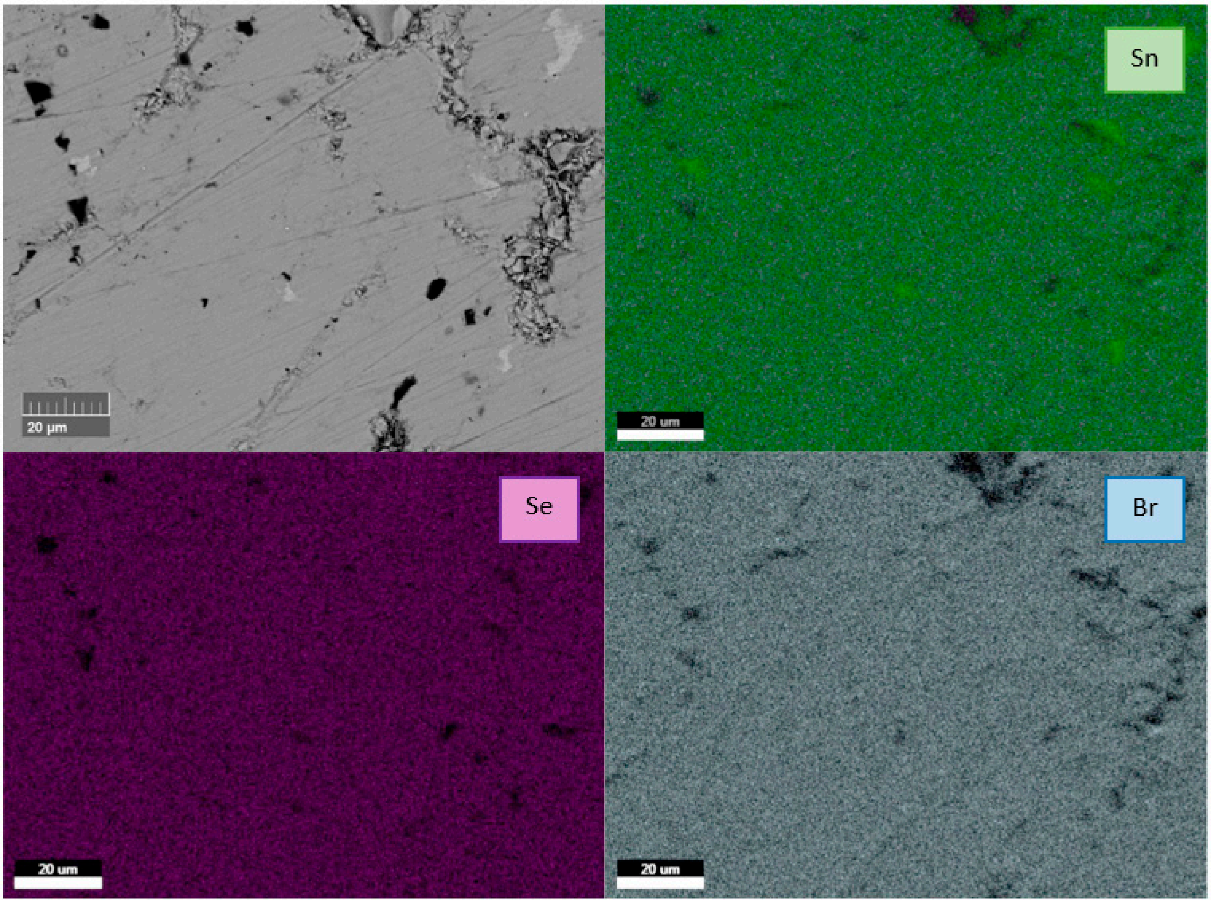

Figure S12 BSE image and corresponding EDX elemental mapping of Br-doped SnSe that was sintered by SPS at 753 K (Br-753) after two weeks of annealing

Table S1 Actual phase composition and density after SPS sintering at different temperatures after two-week annealing

Sample name	EDX results	Density (g/cm^3)
SnSe 573K	$Sn_{0.92(6)}Se_{1.07(4)}$	5.41(9)
SnSe 673K	$Sn_{0.91(8)}Se_{1.08(2)}$	6.09(3)
SnSe 773K	$Sn_{0.92(6)}Se_{1.07(4)}$	5.96(7)
SnSe 873K	$Sn_{0.91(9)}Se_{1.08(1)}$	5.69(3)
SnSeBr 573K	$Sn_{0.87(4)}Se_{1.09(3)}Br_{0.03(3)}$	5.93(1)
SnSeBr 673K	$Sn_{0.91(3)}Se_{1.05(6)}Br_{0.03(1)}$	5.78(6)
SnSeBr 753K	$Sn_{0.91(1)}Se_{1.06(3)}Br_{0.02(6)}$	5.94(1)
SnSeBr 873K	$Sn_{0.90(6)}Se_{1.06(7)}Br_{0.02(7)}$	5.69(1)

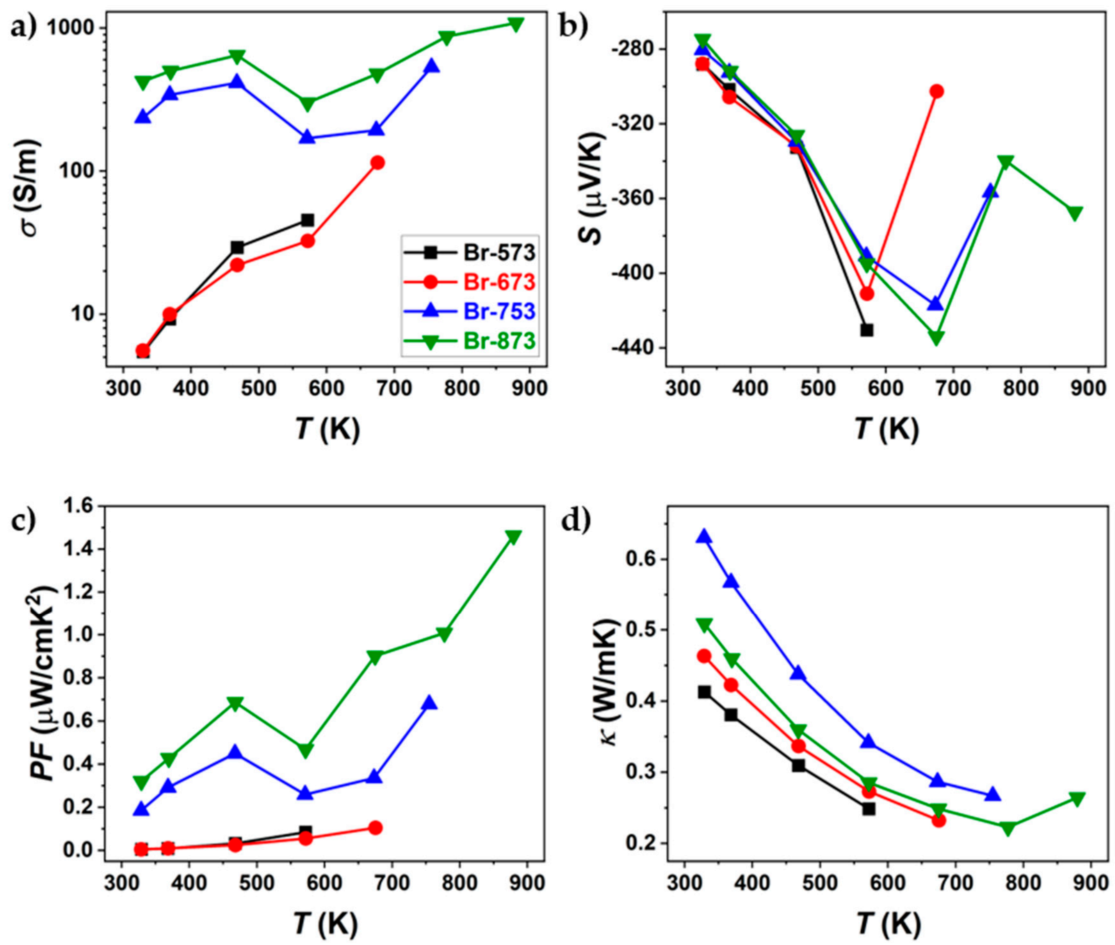

Figure S13 Temperature dependence of a) electrical conductivity, b) Seebeck coefficient, c) Power factor and d) thermal conductivity of Br-doped SnSe, sintered at different temperatures that were all annealed at 753 K
